# Certified Nursing Assistants’ Experiences of Workplace Violence Caring for Persons With Dementia

**DOI:** 10.1093/geroni/igaa057.836

**Published:** 2020-12-16

**Authors:** Chunhong Xiao, Vicki Winstead, Corteza Townsend, Rita Jablonski

**Affiliations:** 1 The University of Alabama at Birmingham, Birmingham, Alabama, United States; 2 University of Alabama at Birmingham, Alabaster, Alabama, United States; 3 UAB School of Nursing, Birmingham, Alabama, United States

## Abstract

Problem: Certified nursing assistants (CNAs) are the primary providers of direct care to persons residing in long term care facilities (LTCFs), many of whom have dementia. The need to deliver direct and intimate care increases CNAs’ exposure to verbal and physical workplace violence. Purpose: To describe CNAs’ experiences of physical and verbal workplace violence experienced during direct care activities in LTCFs. Design: Qualitative. Sample & Procedure: Ten African-American CNAs (9 female, 1 male) were recruited using snowball sampling from multiple LTCFs. Interviews were recorded and transcribed. NVivo12 software was used to manage the thematic analyses. Results: The identified themes were: 1) CNAs’ perception that verbal and physical abuse was “part of the job” and unavoidable; 2) CNAs’ feelings of minimization of the abuse by administration; and 3) inadequate CNA training to recognize and de-escalate triggers of verbal and physical violence, notably care-resistant behavior. Conclusion: The combination of institutional tolerance of workplace violence, coupled with CNAs’ insufficient training in de-escalating volatile interactions with cognitively-impaired residents, is creating an unfavorable, possibly dangerous, workplace environment for CNAs. Implications: As more states elevate assaults on healthcare workers to felony crimes, there is an emerging risk of criminalizing dementia-related behavior in an attempt to address workplace violence. Interventions focused on helping CNAs recognize and de-escalate care-resistant behavior are necessary for violence prevention programs in LTCFs. Limitations: CNAs may have self-censored and under-described the severity of their experiences during face-to-face interviews, even with confidentiality protocols and the practice of off-site interviews.

